# Long-Term Effectiveness and Safety of SGLT-2 Inhibitors in an Italian Cohort of Patients with Type 2 Diabetes Mellitus

**DOI:** 10.1155/2019/3971060

**Published:** 2019-11-04

**Authors:** Maria Mirabelli, Eusebio Chiefari, Patrizia Caroleo, Raffaella Vero, Francesco Saverio Brunetti, Domenica Maria Corigliano, Biagio Arcidiacono, Daniela Patrizia Foti, Luigi Puccio, Antonio Brunetti

**Affiliations:** ^1^Department of Health Sciences, University “Magna Græcia” of Catanzaro, Catanzaro, Italy; ^2^Complex Operative Structure Endocrinology-Diabetology, Hospital Pugliese-Ciaccio, Catanzaro, Italy

## Abstract

**Background:**

SGLT-2 (sodium-glucose cotransporter-2) inhibitors are a novel class of oral hypoglycemic agents for the management of type 2 diabetes mellitus (T2DM). Herein, we aimed to assess the long-term effectiveness and safety of SGLT-2 inhibitors in a Southern Italy population of subjects affected by T2DM.

**Patients and Methods:**

408 diabetic patients treated with one of the three SGLT-2 inhibitors currently available in Italy (dapagliflozin, empagliflozin, and canagliflozin), either alone or in combination with other antidiabetic drugs, were retrospectively assessed at baseline, during, and after 18 months of continuous therapy.

**Results:**

Treatment with SGLT-2 inhibitors resulted in a median decrease in HbA1c of 0.9%, with a percentage of decrement of 12 in relation to the baseline value, followed by a significant reduction (*P* < 0.001) in fasting plasma glucose. Variations in HbA1c occurred independently of the baseline clinical or biochemical characteristics. In addition, treatment with SGLT-2 inhibitors reduced body weight (*P* < 0.008) and decreased diastolic blood pressure (*P* = 0.004). With regard to safety outcomes, 66 patients out of 91 stopped SGLT-2 inhibitors during follow-up because of chronic or recurring genital infections, while the rest experienced other adverse events, such as urinary tract infections, polyuria, nausea, hypotension, dizziness, acute coronary event, worsening of glycemic control status, and rapid deterioration of renal function.

**Conclusion:**

In our patients' population, the glycometabolic effects of SGLT-2 inhibitors were durable and comparable to those observed in multicenter randomized controlled trials. This notwithstanding safety concerns must be raised regarding the frequent occurrence of genitourinary infections and the risk of a rapid decline of renal function in patients with evidence of volume depletion and/or receiving other medications which can adversely affect kidney function.

## 1. Introduction

Over the last four decades, the global prevalence of type 2 diabetes mellitus (T2DM) has quadrupled, in parallel to that of obesity [1], because of a more westernized lifestyle, responsible for most of the excess weight in the modern adult's life [2]. Although with wide regional differences, in 2016, 5.3% of the entire Italian population (16.5% among people aged 65 and over) was affected by diabetes, with a negative record in the Calabrian Region of Southern Italy with ~33% obese people and 8% diabetics [3]. Although adequate glycemic control remains the main therapeutic goal in patients with T2DM, more than half of diabetics do not reach the optimal glycemic target (HbA1c < 7%) recommended by the American Diabetes Association [4], which would significantly reduce the incidence and progression of microvascular complications [5–8].

SGLT-2 (sodium-glucose cotransporter-2) inhibitors are the last class of antidiabetic drugs approved by FDA and EMA regulatory agencies, which could be used in any stage of T2DM, irrespective of comedications. By lowering the renal threshold for glucose excretion, SGLT-2 inhibitors suppress renal glucose reabsorption with insulin-independent mechanisms and are therefore suitable for patients with long-standing diabetes and impairment of *β*-cell function [9]. However, their unique glycosuric mechanism is dependent on the glomerular filtration rate so that in patients with chronic kidney disease (estimated GFR < 45 ml/min/1.73 m^2^), SGLT-2 inhibitors do not increase the urinary glucose excretion and, therefore, are not recommended. Beyond glycemic control, SGLT-2 inhibitors have also the potential of reducing weight, due to the calorie loss through glycosuria, and be beneficial for lowering blood pressure, due to their osmotic diuretic effect [10]. Furthermore, EMPA-REG OUTCOME (Empagliflozin Cardiovascular Outcome Event in type 2 diabetes mellitus) [11–13] and CANVAS (CANagliflozin cardioVascular Assessment Study) [14] have shown that treatment with SGLT-2 inhibitors may reduce cardiovascular morbidity and mortality, as well as the onset and/or progression of nephropathy in high-risk T2DM patients, when compared to standard care. SGLT-2 inhibitors share these glycometabolic and cardio-renal-protective effects with the glucagon-like peptide 1 receptor agonist (GLP1-RA), liraglutide [15, 16]. From a pharmacoeconomics perspective, if the efficacy and safety results of EMPA-REG OUTCOME, CANVAS, and LEADER (Liraglutide Effect and Action in Diabetes: Evaluation of Cardiovascular Outcome Results) were confirmed by real-world evidence, treatment with SGLT-2 inhibitors would become a more cost-effective strategy to achieve glycemic control and prevent cardiovascular death and nephropathy in T2DM patients [17], with the advantage of oral administration. Nonetheless, many questions remain regarding the safety and tolerability of SGLT-2 inhibitors. Clinical trials and preliminary postmarketing research have highlighted the risk of genitourinary infections, urosepsis, diabetic ketoacidosis, volume depletion, and amputation, especially in the most vulnerable categories of patients with diabetes [18–22]. This notwithstanding, so far, only a few observational studies have been conducted on the effectiveness and safety of SGLT-2 inhibitors at a median follow-up of more than one year [23, 24].

The purpose of this study was to evaluate the long-term safety and efficacy of three SGLT-2 inhibitors (empagliflozin, dapagliflozin, and canagliflozin) administered to Calabrian patients with T2DM, attending our endocrinology/diabetology outpatient clinics.

## 2. Patients and Methods

### 2.1. Participants

In this study, we retrospectively analyzed the safety and efficacy of SGLT-2 inhibitors in a Southern Italy population of subjects affected by T2DM. Data were collected from 408 diabetic patients who started treatment with one of the three SGLT-2 inhibitors currently available in Italy: dapagliflozin 10 mg/day (*Forxiga®* and *Xigduo®*), empagliflozin 10 mg and 25 mg/day (*Jardiance®*, *Synjardy®*, and *Glyxambi®*), and canagliflozin 100 mg and 300 mg/day (*Invokana®* and *Vokanamet®*), either alone or in combination with other antidiabetic drugs (including insulin), on the basis of the international clinical practice recommendations for the management of hyperglycemia in T2DM [25]. Participants were recruited from the Operative Units of Endocrinology and Diabetes (AOU “Mater Domini” and the AO Pugliese-Ciaccio in Catanzaro) during the period November 2012 to August 2018, after marketing authorization approval of dapagliflozin (*Forxiga®*) in Italy. Age, sex, body mass index (BMI), blood pressure (BP), lipid profile, fasting plasma glucose (FPG), HbA1c, aspartate aminotransferase/alanine aminotransferase (AST/ALT), serum creatinine, duration of diabetes, micro- and macrovascular complications of T2DM, and any concomitant pharmacological therapy were recorded at baseline for all patients.

### 2.2. Data Collection

Data collection was approved by the ethics committee of *Regione Calabria Sezione Area Centro* (protocol registry number 26 of January 17, 2019). As the data were analyzed anonymously, there was no need for written informed consent. Study was performed in accordance with the Declaration of Helsinki.

### 2.3. Assessments

All patients underwent periodical clinical and biochemical evaluation to monitor the safety and efficacy of SGLT-2 inhibitors therapy. The variables analyzed to assess efficacy included: HbA1c, FPG, body weight, BMI, systolic and diastolic BP, and total daily insulin dose (TDI). Safety variables included fasting lipid profile, AST and ALT liver enzymes, serum creatinine, estimated glomerular filtration rate (eGFR) using both the CKD-EPI creatinine equation and the MDRD study equation, genitourinary infections, hypoglycemic episodes, dehydration, and volume depletion symptoms. Any medical problems, including possible adverse events, were recorded on diary cards, and the entries were reviewed at each study visit.

### 2.4. Outcome Measures

The primary outcome was to test the safety and tolerability of SGLT-2 inhibitors in our patients' population. The primary efficacy outcome measure was the change from baseline in HbA1c after 18 months of treatment. The secondary outcome measures included changes in body weight, BMI, BP, FPG, lipid profile, AST and ALT, TDI, and proportion of participants achieving HbA1c level < 7.0%. In addition, we searched for potential baseline predictors of a better response to therapy.

### 2.5. Statistical Analysis

Initially, each quantitative trait was tested for normality of distribution, using the Shapiro-Wilk normality test. Continuous variables were expressed as median and interquartile range (IQR) and categorical variables as numbers and percentages. The nonparametric Wilcoxon signed-rank test was used for within-group quantitative differences, whereas the two-tailed Fisher's exact test was used for comparisons of proportions. Spearman's rank correlation analysis was used to explore the correlation between safety and efficacy of SGLT-2 inhibitors with clinical and biochemical parameters. A *P* value of <0.05 (two-tailed) was considered statistically significant. Data were analyzed with SPSS 20.0 software (SPSS Inc., Chicago, IL, USA).

## 3. Results

### 3.1. VClinical and Biochemical Baseline Characteristics of SGLT-2 Inhibitor-Treated Patients

A total of 408 diabetic patients started the treatment with SGLT-2 inhibitors. Out of these patients, 246 (60.3%) were treated with empagliflozin, 107 (26.2%) with dapagliflozin, and 55 (13.5%) with canagliflozin.  Table 1 shows the main clinical and biochemical characteristics of these subjects.

### 3.2. Safety of SGLT-2 Inhibitors

**Figure 1:**
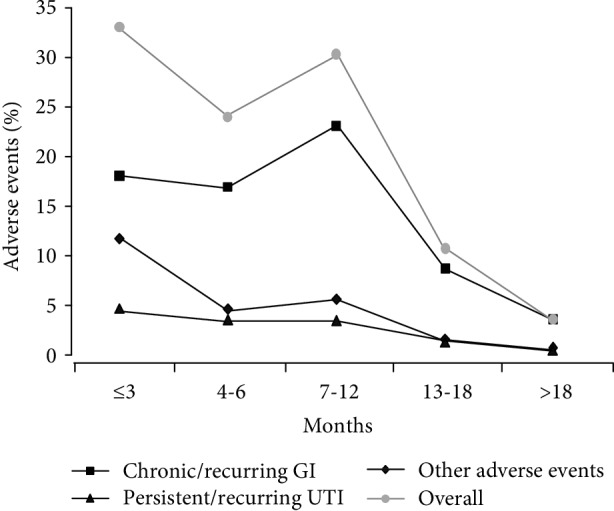
Adverse events. Onset of adverse events over the course of the treatment period. Other adverse events are polyuria, nausea, hypotension, dizziness, acute coronary event, worsening of glycemic control, and rapid deterioration of renal function. GI: genital infections; UTI: urinary tract infections.

27 patients were lost to follow-up after a median duration of 3 months (0-9 IQR), while 98 (24%) individuals (54 females and 44 males) stopped SGLT-2 inhibitors during follow-up because of adverse events. As reported in Table 2, most of them (*N* = 66) discontinued treatment at 7.5 (3-12 IQR) months for chronic or recurring genital yeast infections, whereas 11 patients stopped therapy at 6 (3-12 IQR) months for persistent or recurrent urinary tract infections. 21 patients stopped using SGLT-2 medications for other adverse events, including polyuria (6 patients), nausea (1 patient), hypotension (1 patient), dizziness (1 patient), acute coronary event (1 patient), worsening of glycemic control status (3 patients), rapid deterioration of renal function as defined by a rising of serum creatinine levels (4 patients), and other side effects (4 patients). In 4 individuals, therapy was discontinued for lack of compliance. There were neither lower-extremity amputations nor episodes of stroke during the study period. In terms of percentage, women (31.2%) were more likely to discontinue SGLT-2 inhibitor therapy than men (18.7%) (*P* = 0.005), mainly because of chronic or recurring genital yeast infections (*P* = 0.020) (Table 3). On Spearman's univariate correlation analysis, patients' age was significantly correlated with interruption for side effects (*ρ* = −0.119, *P* = 0.016). No other correlation was detected. As shown in Figure 1, side effects appeared during the first year of treatment, while the three SGLT-2 inhibitors had about the same risk of side effects (Table 4).

### 3.3. Efficacy of SGLT-2 Inhibitors

**Figure 2:**
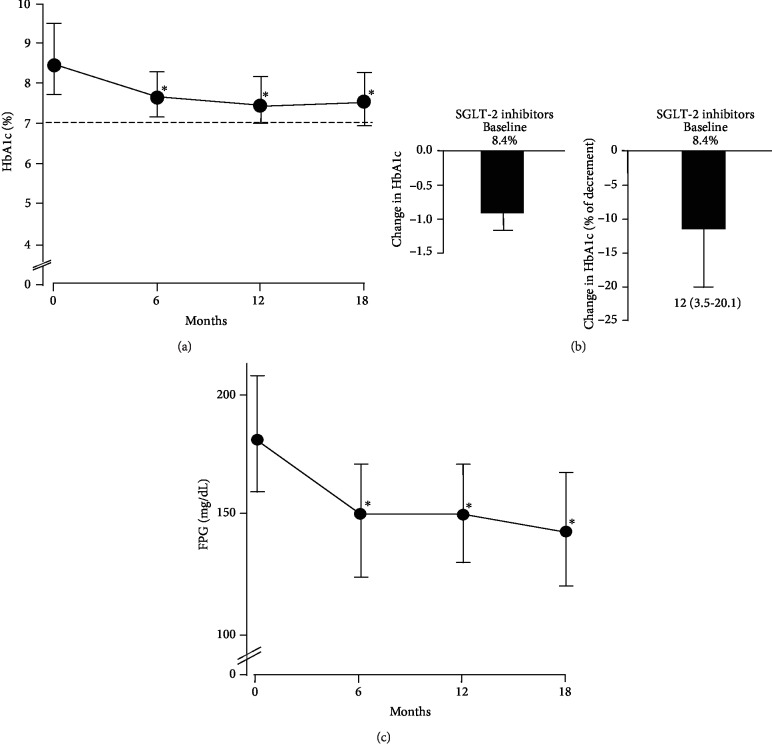
Glycemic efficacy. (a) Trend of HbA1c over the course of the 18-month treatment period. The differences between pre- and posttreatment were evaluated by the Wilcoxon nonparametric test. ^∗^*P* < 0.001 with respect to basal values. (b) Changes in HbA1c from baseline to 18 months, in absolute number (left) and percentage (right). Baseline values are indicated as median. Changes in HbA1c are indicated as median and IQR. (c) Changes in FPG from baseline to 18 months. Baseline values are indicated as median. Changes in FPG are indicated as median and IQR. ^∗^*P* < 0.001.

As the study progressed, we have been able to follow 101 treated patients for at least 18 months. During this 18 months of treatment, a significant reduction in HbA1c was observed over the course of the study period, with a more pronounced decrease in HbA1c during the first six months of drug use (Figure 2(a)). A slight trend towards an increase in HbA1c levels was noted following 18 months of treatment (not shown). Overall, HbA1c decreased from 8.4% (7.7 to 9.4) to 7.5% (6.9 to 8.2) (*P* < 0.001). In details, we observed a reduction of 0.9% (0.3-1.7), with a percentage of decrement of 12 (3.5-20.1) with respect to the baseline value (Figure 2(b)). Spearman's univariate correlation analysis was employed in order to identify the existence of better predictors of response to therapy. However, neither biochemical findings nor clinical determinants of SGLT-2 efficacy were identified.

Considering the secondary outcomes, levels of FPG decreased from 182 mg/dL (160 to 208) to 144 mg/dL (121 to 168) (*P* < 0.001, Figure 2(c)). Also, body weight decreased from 83 kg (75 to 92) to 80 kg (73 to 91) (*P* = 0.008), and this decrease paralleled the reduction in BMI: from 30.2 kg/m^2^ (27.8 to 33.0) to 29.4 kg/m^2^ (26.8 to 32.2) (*P* = 0.009). The decreasing trend in systolic BP, from 135 mmHg (120 to 150) to 130 mmHg (120 to 140), did not reach conventional levels of statistical significance (*P* = 0.111), whereas diastolic BP significantly decreased from 80 mmHg (70 to 80) to 70 mmHg (70 to 80) (*P* = 0.004). Instead, a significant increase emerged in HDL cholesterol after treatment: from 43 mg/dL (36 to 48) to 45 mg/dL (40 to 55) (*P* = 0.004). No significant differences were observed in other serum and urinary parameters, as well as in eGFR and TDI. When determinants of these differences were explored, only the use of sulfonylureas inversely correlated with body weight reduction (*ρ* = −0.551, *P* = 0.001). Finally, 25 further patients (26.3%) reached ADA target for glycemic control (HbA1c < 7%) (31 versus 6, *P* < 0.001). Among them, 11 patients (44%), who were on insulin therapy, held stable or lower daily insulin requirements following combination with SGLT-2 inhibitors: from 26 UI (17 to 32) to 20 UI (10 to 32).

## 4. Discussion

From the results of the present study, it can be concluded that treatment with SGLT-2 inhibitors can significantly lower FPG and HbA1c in T2DM patients over 18 months of follow-up in real-life clinical practice. As seen in our case, patients using SGLT-2 inhibitors showed a reduction in HbA1c of about 1% at the end of the study period, and over a quarter of participants achieved the ADA glycemic targets, thus closely resembling the efficacy results of randomized clinical trials with these agents [26, 27]. Improvement in glycemic control was independent of baseline HbA1c levels, BMI, and other clinical and biochemical parameters, indicating that treatment with SGLT-2 inhibitors can be initiated in diabetic patients independent of the duration of diabetes and the baseline levels of HbA1c. In our patients, apart from durability of glycemic efficacy, SGLT-2 inhibitors provided weight loss, as well as a considerable reduction in diastolic BP and a slight declining tendency for systolic BP, thereby supporting the pleiotropic effects of this novel class of antidiabetic agents [10, 28]. However, our results about BP are partially inconsistent with those reported in recent meta-analyses of clinical trials, which demonstrate modest reductions in both systolic and diastolic BP with the use of SGLT-2 inhibitors [29–31]. Differences in background antihypertensive regimens, small sample sizes, short follow-up periods, methods for assessing BP, and assessment of antihypertensive effects as secondary outcomes in most studies may explain these discrepancies. Also, discrepancies might arise from population-specific genetic heterogeneity, which may influence distinct BP variations in response to environmental or pharmacological interventions [32].

Only the concomitant use of SGLT-2 inhibitors with sulfonylureas was inversely correlated with weight reduction, making this combination therapy less suitable for overweight and obese patients as it may encourage weight gain [33]. Also, for the first time in a real-world setting, we investigated the long-term impact of SGLT-2 inhibitors on insulin therapy, showing no differences in TDI up to 18 months treatment. The tendency to maintain constant TDI up to 18 months, with significant improvement in both glycemic control and weight loss, should be considered when assessing T2DM patients on insulin therapy.

Consistent with previous studies [29], our findings indicate that SGLT-2 inhibitors cause a modest but significant increase in HDL cholesterol, with no effect on serum creatinine levels and eGFR. These findings complement those of the landmark cardiovascular outcomes trial on empagliflozin [13], in which the eGFR remained fairly stable through 6 years of follow-up, while gradually declining with placebo. A major decline in renal function occasionally occurs during SGLT-2 inhibitor therapy, often associated with specific coadministered medications. In March 2016, the FDA reinforced the existing warning on the potential risk of acute renal failure with SGLT-2 inhibitors, following numerous postmarketing case reports [34]. This adverse event is probably the expression of the intrarenal hemodynamic changes occurring during the first weeks of treatment with SGLT-2 inhibitors, which result in a transient eGFR reduction [35–37]. These intrarenal hemodynamic changes may be accentuated in susceptible patients, as a consequence of volume depletion induced by glucose-induced osmotic diuresis [38], reduced angiotensin II-mediated efferent arteriolar vascular tone [39], and concurrent use of nonsteroidal anti-inflammatory drugs or radiocontrast agents [40].

With regard to safety issues with SGLT-2 inhibitors, our data are in contrast to other postmarketing observational studies [23, 41]. Chronic or recurring genital yeast infections were the main cause of treatment discontinuation in our patient population, with a clear female preponderance. Most infectious events occurred within the first year of treatment, in line with a pooled analysis of phase III clinical studies [22]. SGLT-2 inhibitor-treated patients improved glycemic control with respect to baseline, supporting the idea that genitourinary tract infections were not correlated with poorly controlled hyperglycemia [42], but rather with increased urinary glucose excretion, that may enhance *Candida* colonization of genital tissues [43] and the growth rate of potential uropathogens [44].

One strength of the present study is the large subset of adult patients with T2DM who received at least one dose of SGLT-2 inhibitors from a single tertiary care center, regardless of previous and/or concomitant antidiabetic agents, duration and severity of disease, comorbidities, and follow-up time. The study reflects real-life clinical practice, partially avoiding the selection bias that is common in randomized clinical trials. All patients were recruited in Calabria, Southern Italy, a region with limited genetic diversity [45], which lowers interindividual variability among people, including variability in drug response [46]. Improved long-term efficacy outcomes following treatment with the GLP-1RA, liraglutide, were reported by us before, in this region, in terms of reduced HbA1c, FPG, body weight, and systolic BP, throughout a follow-up period of 18 months [47]. For the first time, herein, we focused on the long-term effectiveness of SGLT-2 inhibitors. However, caution should be taken when evaluating the degree of benefit from SGLT-2 inhibitors in this study, owing to its retrospective observational nature.

## 5. Conclusions

Our study indicates that the beneficial durable effects of SGLT-2 inhibitors in Calabrian patients with T2DM are comparable to those from multicenter randomized controlled trials. Safety concerns must be raised regarding the occurrence of genitourinary infections, which, although generally mild or moderate in intensity, tend to recur and eventually lead to treatment discontinuation. A warning should also be issued about the risk of rapid deterioration of renal function in patients prone to volume depletion and/or receiving medications, such as nonsteroidal anti-inflammatory drugs and renin-angiotensin-aldosterone system blockers. So, it is recommended that renal function should be monitored closely during SGLT-2 inhibitor therapy.

## Figures and Tables

**Table 1:** Baseline clinical and biochemical characteristics of all 408 T2DM patients treated with SGLT-2 inhibitors.

Baseline features	*N* (%)
Female gender	173 (42.4)
Age (years)	62 (55-68)^a^
Diabetes duration (years)	12 (7-19)^a^
Diabetes duration ≥10 years	254 (62.3)
Body weight (kg)	83 (74-92.5)^a^
BMI (kg/m^2^)	30.1 (27.2-36.6)^a^
BP (mmHg)	
Systolic	130 (120-146)^a^
Diastolic	80 (70-80)^a^
FPG (mg/dL)	180 (152-213)^a^
HbA1c (%)	8.3 (7.6-9.6)^a^
Serum creatinine (mg/dL)	0.8 (0.7-0.95)^a^
eGFR (ml/min/m^2^)	90.6 (77.8-105.3)^a^
Total cholesterol (mg/dL)	166 (141.5-191)^a^
HDL-C (mg/dL)	42 (35-50)^a^
Triglycerides (mg/dL)	142 (98.2-191.5)^a^
AST (UI)	23 (19-32)^a^
ALT (UI)	24 (19-34)^a^

Comorbidities	

Hypertension	333 (81.6)
Coronary artery disease	78 (19.1)
History of stroke/TIA	13 (3.2)
Peripheral artery disease	21 (5.1)
Obesity (BMI ≥ 30 kg/m^2^)	210 (52.2)
Overweight (BMI ≥ 25 kg/m^2^)	147 (36.7)
Other comorbidities	264 (64.7)
Diabetic microvascular complications	160 (39.2)
Diabetic retinopathy	88 (21.6)
Early diabetic nephropathy	47 (11.5)
Overt diabetic nephropathy	25 (6.1)
Diabetic neuropathy (autonomic/peripheral)	55 (13.5)

Concomitant medications	

ACE inhibitors	129 (31.6)
Angiotensin II receptor blockers	149 (36.5)
Calcium channel blockers	90 (22.1)
Beta blockers	117 (28.7)
Diuretics	117 (28.7)
Loop diuretics	33 (8.1)
Alpha 1 blockers	21 (5.1)
Statins	241 (59.1)
Ezetimibe	24 (5.9)
Cardioaspirin	111 (27.2)
NSAIDs	13 (3.2)
Metformin	320 (78.4)
Sulphonylureas	49 (12.0)
Meglitinides	38 (9.3)
DPP-4 inhibitors	14 (3.4)
GLP-1 receptor agonists	12 (2.9)
Pioglitazone	9 (2.2)
Acarbose	6 (1.5)
Insulin	238 (58.3)

^a^Median (IQR) values. BMI: body mass index; BP: blood pressure; FPG: fasting plasma glucose; HbA1c: glycated hemoglobin; eGFR: estimated glomerular filtration rate (MDRD formula); HDL-C: high-density lipoprotein-cholesterol; ALT: alanine aminotransferase; AST: aspartate aminotransferase; NSAIDs: nonsteroidal anti-inflammatory drugs; DPP-4: dipeptidyl peptidase 4; GLP-1: glucagon-like peptide 1.

**Table 2:** Reasons and time for treatment discontinuation in patients who were treated with SGLT-2 inhibitors.

	Patients, *N* (%)	Months, median (IQR)
Chronic or recurring genital yeast infections	66 (67.4)	7.5 (3-12)
Persistent or recurring urinary tract infections	11 (11.2)	6 (3-12)
Other adverse events^a^	21 (21.4)	3 (1-9)
Overall	98 (100)	6 (2-12)

^a^Polyuria, nausea, hypotension, dizziness, acute coronary event, worsening of glycemic control, and rapid deterioration of renal function.

**Table 3:** Gender differences in treatment interruption due to adverse events.

	Females, *N* = 173 (%)	Males, *N* = 235 (%)	*P* value
Chronic or recurring genital yeast infections	37 (21.4)	29 (12.3)	0.020
Persistent or recurring urinary tract infections	7 (4.0)	4 (1.7)	0.216
Other adverse events^a^	10 (5.8)	11 (4.7)	0.655
Overall	54 (31.2)	44 (18.7)	0.005

^a^Polyuria, nausea, hypotension, dizziness, acute coronary event, worsening of glycemic control, and rapid deterioration of renal function. Statistical analysis was performed using Fisher's exact test.

**Table 4:** Interdrug differences in terms of adverse events.

	Empagliflozin, *N* = 62 (%)	Dapagliflozin, *N* = 25 (%)	Canagliflozin, *N* = 11 (%)
Chronic or recurring genital yeast infections	41 (66.1)	16 (64.0)	9 (81.8)
Persistent or recurring urinary tract infections	7 (11.3)	3 (12.0)	1 (9.1)
Other adverse events^a^	14 (22.6)	6 (24.0)	1 (9.1)

^a^Polyuria, nausea, hypotension, dizziness, acute coronary event, worsening of glycemic control, and rapid deterioration of renal function.

## Data Availability

The data used to support the findings of this study are included within the article.
